# Studying Axon-Astrocyte Functional Interactions by 3D Two-Photon Ca^2+^ Imaging: A Practical Guide to Experiments and “Big Data” Analysis

**DOI:** 10.3389/fncel.2018.00098

**Published:** 2018-04-13

**Authors:** Iaroslav Savtchouk, Giovanni Carriero, Andrea Volterra

**Affiliations:** Department of Fundamental Neurosciences, University of Lausanne, Lausanne, Switzerland

**Keywords:** astrocytes, axons, VoI, ImageJ plugin, 5D datasets, calcium signaling, volumetric imaging, 3D calcium imaging

## Abstract

Recent advances in fast volumetric imaging have enabled rapid generation of large amounts of multi-dimensional functional data. While many computer frameworks exist for data storage and analysis of the multi-gigabyte Ca^2+^ imaging experiments in neurons, they are less useful for analyzing Ca^2+^ dynamics in astrocytes, where transients do not follow a predictable spatio-temporal distribution pattern. In this manuscript, we provide a detailed protocol and commentary for recording and analyzing three-dimensional (3D) Ca^2+^ transients through time in GCaMP6f-expressing astrocytes of adult brain slices in response to axonal stimulation, using our recently developed tools to perform interactive exploration, filtering, and time-correlation analysis of the transients. In addition to the protocol, we release our in-house software tools and discuss parameters pertinent to conducting axonal stimulation/response experiments across various brain regions and conditions. Our software tools are available from the Volterra Lab webpage at https://wwwfbm.unil.ch/dnf/group/glia-an-active-synaptic-partner/member/volterra-andrea-volterra in the form of software plugins for Image J (NIH)—a de facto standard in scientific image analysis. Three programs are available: *MultiROI_TZ_profiler* for interactive graphing of several movable ROIs simultaneously, *Gaussian_Filter5D* for Gaussian filtering in several dimensions, and *Correlation_Calculator* for computing various cross-correlation parameters on voxel collections through time.

## Introduction

### Understanding the role of astrocytes in brain network computation

Astrocytes are highly complex cells, interacting with all the other cell types in the CNS, and executing a myriad of functions, including support, homeostatic, and signaling functions (Araque et al., 2014). While there exists a gross understanding of the role(s) of astrocytes in these functions, the exact underlying mechanisms are less clear. One area of intense investigation is whether astrocytes sense and respond to synaptic activity. At what levels are astrocytes recruited: can they detect even a single-synapse release event or do they sense just the synchronized activity of thousands of synapses? Understanding if and how astrocytes respond (in terms of Ca^2+^ elevations) to different levels of neuronal inputs is important in order to understand the conditions under which these cells can signal back to synapses, e.g., by Ca^2+^-dependent exocytosis of chemical mediators (gliotransmitters), and therefore the spatial and temporal frames of their effects on synaptic functions (Araque et al., 2014; Rusakov, 2015).

Initially, astrocytes in culture and brain slices were found to respond to externally applied neurotransmitters with concentration-dependent Ca^2+^ elevations (Cornell-Bell et al., 1990; Porter and McCarthy, 1997). Subsequently, studies focused on synaptically-evoked astrocytic Ca^2+^ responses, but results in this case were contrasted, with several groups reporting an astrocytic detection at the level of a single action potential (AP), while others only after repeated stimulation such as with high-frequency, high-pulse-count trains (HFS). Determining which of the two reported sensitivities, the high or the low, corresponds to the true sensitivity of astrocytes is crucial, as it directly impinges upon the kind of information that astrocytes can process and their role in brain network computation, which is at present a matter of strong debate (Bazargani and Attwell, 2016; Savtchouk and Volterra, 2018).

Studies finding *high* astrocytic sensitivity to neuronal activity could show responses to a single or few APs. This evidence was obtained either directly, by evoking APs via minimal electrical axonal stimulations while monitoring in parallel local astrocytic responses with a Ca^2+^ indicator (Grosche et al., 1999; Matyash et al., 2001; Panatier et al., 2011; Honsek et al., 2012; Sun et al., 2014), or indirectly, by observing a reduction in endogenous local astrocytic Ca^2+^ signals upon pharmacological blockade of spontaneous sparse APs (Di Castro et al., 2011). In contrast, studies finding *low* astrocytic sensitivity claimed that reliable astrocytic Ca^2+^ elevations could not be observed in response to individual stimulations but only to multiple stimulations evoked electrically or through cutaneous stimulation, both *in vitro* (Haustein et al., 2014; Jiang et al., 2016) and *in vivo* (Nizar et al., 2013). For example, one such study reported that astrocytes respond to as few as 8–15 pulses reliably, whereas they respond to a lower number of pulses with reduced reproducibility (Haustein et al., 2014).

The above contrasting data highlight not only the importance but also the methodological challenge of precisely determining the synaptic response properties of astrocytes. For instance, studies using minimal axonal stimulations had to place electrodes very close to the probed astrocyte due to the unknown and hardly predictable nature of axon paths and of their contact points with astrocytes in the hippocampus (see **Figure 4B** and Bindocci et al., 2017). However, this setting carries the risk of inducing direct voltage effects onto the astrocyte membrane, a point of concern since astrocytes are known to respond to local electrical stimulation with Ca^2+^ elevation in a neuron-independent manner (Schipke et al., 2002). On the other hand, stimulations performed from remote sites, while safer, have other important drawbacks and likely underestimate the astrocytic responses particularly at low levels of stimulation. This because of the small size and unpredictable location (even outside the selected two-photon focal plane) of the responding astrocytic volume with respect to the stimulation site, and of the difficulty of teasing apart the evoked responses from the endogenous Ca^2+^ activity ongoing in the astrocyte. Crucially, by combining monitoring of a large 3D astrocytic volume to stimulation protocols and analyses allowing for reliably sorting of evoked and spontaneous astrocytic responses, we were able to overcome the above difficulties and reach a solid conclusion about the correlated nature of neuronal and astrocytic signals. All of this was accomplished in a recently published study (Bindocci et al., 2017), for which we here provide the detailed guide and the relevant software plugins.

### Emerging need for analysis tools for large imaging datasets (“big data”)

In our study (Bindocci et al., 2017), we decided to apply 3D Ca^2+^ imaging to astrocytes. One of the consequences was the generation of large microscopy datasets (the so-called “big data”). Big data production is an ongoing trend in neuroscience in general, because by them one obtains unprecedented levels of information on the spatiotemporal positioning and interactions between the key brain cell components. Large speed/scale functional Ca^2+^ imaging in particular is known to generate datasets of many gigabytes to terabytes, which represents a practical challenge for both initial exploration and automated analysis of the experimental data. At this scale, manual identification and processing of each Region of Interest (ROI) is time consuming, and therefore automated detection and analysis of individual functional *units* and signals is needed.

In this context, handling and analysis of big data concerning neuronal activity can rely on a number of already existing tools. Several software suites have been developed. Some use threshold- and morphology-driven approaches (Srinivasan et al., 2015; Agarwal et al., 2017). Others are largely centered on applying linear algebra/singular-value decomposition (SVD)/matrix factorization approaches (PCA/ICA, CNMF) (Mukamel et al., 2009; Pnevmatikakis et al., 2016; Grewe et al., 2017). Their universal goal is to identify independent units (in the neuronal context, typically active somas) and to extract the corresponding signals. These techniques have recently achieved the ability to (semi)automatically detect and extract somatic activity signals from Ca^2+^ datasets on the scale of hundreds to thousands of neurons at levels comparable to manually-curated datasets (Freeman et al., 2017; Berens et al., in review)—see e.g., CNMF (Pnevmatikakis et al., 2016) or Suite2P (Pachitariu et al., in review). Despite their power, these techniques still rely on a number of fundamental assumptions in both their mathematical underpinnings (consult e.g., Shlens, 2014) and practical application. Some general assumptions are that: (1) a single well-defined unit such as a soma carries most of the relevant information (e.g., on action potential firing); consequently, it becomes generally desirable that somatic ROI detection is optimized and activity of other sub-cellular components (dendrites, neuropil, axons) is instead minimized by subtraction; (2) constraints in terms of size and/or shape can be imposed to the ROI-defined structure; (3) each active pixel belonging to such structure follows the same time-course as the entire structure; (4) generally, the ROI-defined structure is repeatedly and reproducibly activated multiple times throughout the recording; (5) Ca^2+^ events have stereotyped kinetics mostly dependent on the properties of the dye used (with certain rise/decay-time characteristics considered as genuine); (6) background noise can be accurately modeled and discarded, be it coming from true noise sources or the surrounding neuro(glia)pil.

These assumptions are appropriate when applied to the study of neuronal firing activity, but they can spectacularly break down for astrocytes, which lack a clear morpho-functional specialization (no action potentials at the soma!) and exhibit a wide spatio-temporal diversity of Ca^2+^ responses, which also tend to lack stereotyped kinetics. Direct application to astrocytes of the analysis tools developed for neuronal population-level analysis can therefore be inappropriate. As indicated, such tools generally expect signals to derive from somatic regions. However, in astrocytes monitoring the soma activity alone does not capture the full output of the cell. Quite the contrary: the absolute majority of astrocytic Ca^2+^ transients are located outside the soma, nominally in astrocytic main processes, end-feet, and optically sub-resolved fine processes, globally termed the “gliapil” (Bindocci et al., 2017), while independent units (or “microdomains”) within astrocytes may dynamically merge and split under some circumstances.

### Software tools for analyzing astrocytic signals

Shape, size, location, and duration of astrocytic Ca^2+^ responses are highly variable. This translates into the need for a larger hands-on participation of human experimenters in analysis of the astrocytic Ca^2+^ data than in the case of neuronal somatic Ca^2+^ dynamics, and creates a need for more exploratory-based interactive tools, with fewer underlying assumptions and an extra capability of providing exploratory visualization in real time. An example of such toolset is the one that we recently developed for analysis of our 3D two-photon imaging data of spontaneous and evoked Ca^2+^ activity in astrocytes (Bindocci et al., 2017).

Our 3D imaging approach involves fast, high-resolution sampling of a cuboid volume through time and across multiple detector channels (in reality, 5-D: 3D volume + time + color information) and generates datasets of ~25–50 GB per single experiment. Volumetric Ca^2+^ imaging is technically accomplished by increasing the scan rate for a single plane while, at the same time, increasing the speed of vertical objective re-positioning between planes (Gobel et al., 2007; Grewe et al., 2010, 2011; Kampa et al., 2011; Katona et al., 2012; Fernandez-Alfonso et al., 2014; Bindocci et al., 2017). For this, instead of classical galvanometers, we used an AOD or a resonant scanhead coupled to a piezo-actuated z-drive for the objective (Bindocci et al., 2017). This way, the dwell time of each voxel is greatly reduced, leading to a significant increase in the total voxel scan rate with the final result of capturing multiple focal planes in the time it normally takes to acquire a single plane. While the storage requirements for the 3D datasets are substantial, a main difficulty is the lack of user-friendly analytical tools allowing for interactive exploration of the datasets, such as a real-time display of Ca^2+^ traces from the volumes of interest interactively defined and moved by the user. It is the kind of tools that we developed for our study and present here below.

### Inherent limitation of ROI-based approaches for astrocyte data processing

Before we discuss the new tools, it is important to point out the limitations that have emerged in the approaches used so far for analysis of astrocyte Ca^2+^ dynamics. Coming back to the tools setup for handling neuronal Ca^2+^ big data, as indicated, their typical approach consists of complexity reduction, accomplished by placing large ROIs onto neuronal somas, thereby merging information from a large number of voxels into a single trace, either manually, or via linear algebra-based approaches (see e.g., Grewe et al., 2017). In such a way, millions of discrete time-series (one for each voxel) are efficiently transformed into mere tens or hundreds, permitting efficient storage and processing. While the unique voxel information is lost, this is mitigated by choosing ROIs in such a way that these purportedly correspond to the underlying biological units of activity (or alternatively, computing voxel groups that, after clustering, preserve the maximum amount of variance). In a sense, the strategy is valid for neurons, as Ca^2+^ events at the neuronal soma do correctly report the AP firing activity of the neuron, particularly when a genetically encoded Ca^2+^ indicator (GECI) such as GCaMP6 is used, capable of discriminating individual APs. Nonetheless, even for neurons, the strategy of ROI-based complexity reduction appears to work well only for studies looking at the firing of neuronal population ensembles (with each soma/neuron treated as a quantal firing unit that can overlap—but not dynamically merge and split up—with another unit). This approach is, however, not appropriate when the goal is to understand local neuronal processing, such as dendritic Ca^2+^ computation (Branco et al., 2010). In cases like this, tens to hundreds of ROIs should be in principle defined for each region of the cell, typically at locations that are thought to themselves act as functional units, such as individual spines, including also the inactive or sparsely active structures!

Analytically, the situation in astrocytes is similar to that in dendrites, with the major issue that there is no solid basis for defining what a quantal functional unit is. A typical astrocyte can contact tens to hundreds of thousands of synapses. Contacts mostly occur at the level of thin (~100–200 nm diameter) peri-synaptic astrocytic processes [PAPs, (Chao et al., 2002) cited in (Shigetomi et al., 2013; Volterra et al., 2014)], which represent the peripheral branches and sub-branches of the core processes stemming from the soma of the cell. Such fine structures, the gliapil, are typically below optical resolution limit and appear in two-photon imaging as a sub-resolved fuzzy *cloud* surrounding the astrocytic core. Nonetheless, they are most likely where the local astrocyte-synapse functional interactions take place and the source of the corresponding local astrocytic Ca^2+^ elevations, i.e., the microdomain (or nanodomain) functional units (reviewed in Araque et al., 2014; Volterra et al., 2014; Rusakov, 2015). Early studies, however, attempted at understanding astrocytic Ca^2+^ activity and synaptic interactions by typically placing ROIs at the somas of astrocytes (e.g., Wang et al., 2006), probably by analogy to neuronal studies. Over the years, detailed investigations of Ca^2+^ activity in astrocytes using high spatial-resolution two-photon imaging led to progressively uncover a wide diversity of Ca^2+^ transients in astrocytes, with many tiny non-propagating (local) events happening in the processes and gliapil (Di Castro et al., 2011; Haustein et al., 2014). These studies also established that somatic Ca^2+^ transients represent a small minority (≤ 3–5%) of the total activity of the astrocyte (Bindocci et al., 2017). In this situation, a comprehensive look at the entire structure (volume) of the astrocyte is required in order to understand the properties of the astrocytic responses, and ROI-based or SDV-related approaches face inherent limitation.

**Figure 1:**
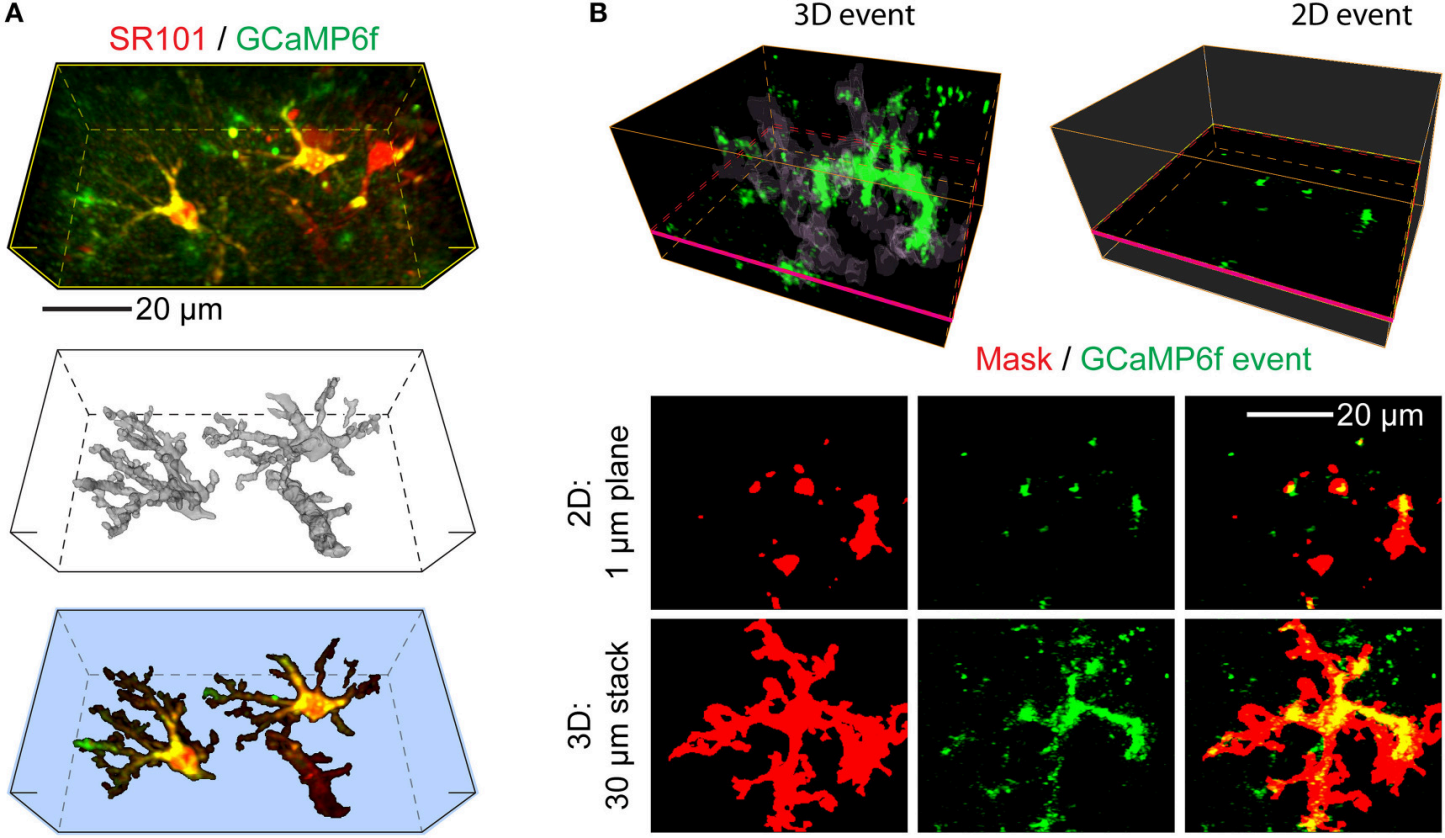
Advantages of 3D Ca^2+^ imaging in astrocytes over 2D-based techniques. Applying 2D-based approaches to astrocytes is likely to produce artifacts, such as incomplete and incorrect capture of the structural and functional information. **(A)** An example of a 3D volume selected for imaging, which contains several astrocytes expressing GCaMP6f calcium indicator. *Top*: average projection of the morphological (SR101 dye, red, labels astrocytes) and functional indicators (GCaMP6f, green, transgenically expressed in astrocytes). *Middle*: an SR101-based reconstruction (mask) of the *core* borders of the two GCaMP6f expressing astrocytes present in the selected imaging volume. Smaller processes and most of the “gliapil” are not included here. A blood vessel is also labeled via the astrocytic end-feet. *Bottom:* The same stack as at the top, segmented based on the 3D reconstruction. **(B)** A representative example of GCaMP6f activity as seen in 3D (*Top left*) by monitoring the volume of an astrocyte (average of 2 s, thresholded to 4 SD) or in 2D (*Top right*), by monitoring an individual focal plane. The regions segmented by the astrocytic *core* mask are faintly outlined, magenta rectangle indicates the location of the selected focal plane. A large Ca^2+^ event is apparent in a vertical process in the 3D, but not 2D situation. By using a single focal plane, 2D imaging in practice fragments the 3D structure of the astrocyte and misses most of it. The majority of the large Ca^2+^ event apparent in 3D is likewise missed and fragmented into multiple smaller events, generating artifacts in reporting the event frequency and spatial properties. *Bottom*: A top-down projection of 30 individual focal planes (3D) recovers the astrocyte structure fragmentation and most of the missing Ca^2+^ event allowing correct reporting of its properties, as compared to a single 2D plane.

Over the years, this new understanding of astrocyte Ca^2+^ dynamics led to a parallel progress in astrocytic Ca^2+^ data analysis. Researchers started to appreciate the inherent limitations of ROI-based methods, notably of arbitrary sizing and placement, and attempted to overcome these in a number of ways. One approach was to systematically split the core structure of astrocytes into smaller blocks and analyze each of these individually, as was done (e.g., Di Castro et al., 2011). This led to the identification of small, fast, and surprisingly local Ca^2+^ transients (dubbed “focal events”) in the processes of astrocytes. Other approaches focused on automated generation of ROIs based on the detected activity (Srinivasan et al., 2015; Agarwal et al., 2017). Under ideal circumstances, these new activity-driven ROI approaches should generate individual ROIs that correspond to individual functional microdomains (i.e., the functional units), but in practice, they are very likely to result in incorrect merging or splitting of the functional domains into arbitrary ROIs because of a number of problems (see Figure 1). A major one is that ROIs were generated in 2D, while the underlying structure (and activity) of astrocytes is three-dimensional (Figure 1A). Use of conventional two-photon acquisitions restricted to an individual focal plane makes it just not technically feasible to both sample large volumes in 3D and generate the multi-plane ROIs. Even in 2D, there were significant problems with automatically generated ROIs often mismatching the real locations of the truncated 3D signals. In particular, because microdomain detection was based on time-projected activity maps (Srinivasan et al., 2015), multiple overlapping domains could be merged into a larger apparent ROI, as well as single domains split up into multiple ROIs. The overlap issue could in principle be corrected by looking at individual frames for event detection (Wu et al., 2014; Bindocci et al., 2017), but splitting up of single events into multiple fragments remains an inherent technological limitation due both to signal-to-noise ratio (SNR) issues and to using 2D imaging methods to study 3D signals. Another main problem comes from most of the astrocytic Ca^2+^ activity occurring in the optically sub-resolved regions, the gliapil, which account for ~80% of an astrocyte volume. Extending analysis to these regions is therefore crucial, albeit far from straightforward, due to lack of any reliable structural information. To tackle the problem, we developed an approach based on limiting analysis to identifying individual active voxels with no pretention of merging them into functional domains (Bindocci et al., 2017). While this can be considered a conservative approach, it is still useful to quantify activity throughout an astrocyte and does not incur in mistakes, which are unavoidable when trying to arbitrarily merge active voxels from sub-resolved regions.

### Understanding neuron-astrocyte computation: a question of scale

With the above issues in mind, a next level of challenge comes when one wants to relate activity at neuronal synapses and in astrocytes. In order to understand the cause-effect relations between these two types of activity, analysis needs to be able to extract and link together in time and space functionally meaningful signals generated by the two cell types. To address this, a relatively straightforward approach is cross-correlation analysis. Early attempts to analyze astrocytic Ca^2+^ responses using such correlation approaches (Tashiro et al., 2002) suggested little direct link between astrocytic Ca^2+^ transients and neuronal discharges. However, such historical studies were hampered by many of the limitations already discussed, notably they were performed with 2D imaging and were based on arbitrary placement of ROIs in somas for both neurons and astrocytes. Moreover, due to the dye loading techniques used at the time, they could be performed only in very young slices (below P15), where astrocytic and synaptic activities are not fully representative of the situation in the adult (Sun et al., 2013). Later investigations by the same group, using much older mice, improved indicators, and finer-graded ROIs, have instead reported a clear link between astrocytic Ca^2+^ oscillations and cortical neuron up-states (Poskanzer and Yuste, 2016).

### New analytical tools

With rapid raster-scan multi-dimensional (3D + time) two photon imaging becoming increasingly practical nowadays because of several technological innovations (faster scanners, better indicators, higher-sensitivity detectors, and faster electronics) that trade off voxel dwell time for an overall increase in speed, the demand for free, open tools for 3D analysis is rapidly increasing in parallel. We have contributed to the still limited offer by developing and releasing [see Supplementary Materials (Presentation 1.zip)] (https://wwwfbm.unil.ch/dnf/group/glia-an-active-synaptic-partner/member/volterra-andrea-volterra three free plugins that expand the built-in hyperstack analysis functionality of the freely available ImageJ program (NIH) (Schneider et al., 2012):

1. *Gaussian_Filter5D:* a simple 5D Gaussian filter plugin for signal processing. This was currently missing from the default ImageJ tools, since the built-in 3D filter functionality does not fully support filtering higher-order stacks (such as combined z+t). The current plugin is natively capable of processing a 5-D stack, and is able to filter in all dimensions (except across channels). A *DC offset* option is provided to blank arbitrary noise values below a selectable threshold.
2. *MultiROI_TZ_profiler*: a multi-ROI trace plotter allowing for interactive, real-time exploration of the 5D data by the user. This expands the functionality of the built-in “*Z-axis profiler*” tool set up by the NIH developers (Baler and Rasband, 2003) which allows for plotting the average ROI signal through space or time. Using the *Z-axis profiler* code as a starting base, we added the multi-ROI (multi-trace) capability, as well as the capability of adding an externally loaded trace (e.g., an electrophysiology trace) to the plot. A number of other user-friendly options such as filtered trace overlay, color-coding and various display normalization options, are also provided via an extra interface window. The plugin implements dynamic update capability, allowing the user to move and modify ROIs, change channels and focal planes, and see the resulting trace changes in real time.
3. *Correlation_Calculator*: a cross-correlation plugin that performs a comprehensive list of voxel-by-voxel calculations through time in search of temporal correlation versus either an externally loaded trace, a binarized stimulus waveform, or an individual ROI extract. This allows for the easy identification of putatively time-locked regions within a 3D imaging volume. To improve the performance under the jittering response conditions, cumulative cross-correlation (area under the curve) is available in addition to the normal peak calculation. In our studies, we have applied this plugin to axonal stimulation paradigms (minimal stimulations) in the dentate gyrus and have thereby identified several regions within an astrocyte that reliably responded to the stimuli. Such regions corresponded to a fraction of <1% of the total analyzed astrocytic volume (Bindocci et al., 2017).

## Methodological aspects: a practical guide

In this section, we provide a practical guide based on our experience as to how Ca^2+^ transients evoked in astrocytes by neuronal activity can be recorded via 3D two-photon Ca^2+^ imaging and subsequently analyzed. We discuss the type of equipment required, the experimental procedures for expressing GECI in neurons and astrocytes, for stimulating axons and for Ca^2+^ imaging in brain slices or *in vivo* in awake mice, as well as the tools to be used for data storage, handling and analysis.

### Reagents, equipment, and axonal stimulation protocols

#### Reagents

- Distilled, deionized electrophysiology-grade water (18.2 MOhm-cm)
- aCSF, prepared according to a number of possible recipes, e.g., as follows (in mM): 118 NaCl, 2KCl, 2 MgCl_2_, 2 CaCl_2_, 25 NaHCO_3_, 1.2 NaH_2_PO_4_ and 10 glucose
- Borosilicate glass electrodes
- Fluorescent quantum dots (e.g., 540 nm), toluene, and methanol for coating the stimulation pipette.

#### Equipment

*Imaging system*: in principle, this can be any system providing sufficient space-time resolution and sensitivity appropriate to the question at hand. In our case, for studying astrocytic Ca^2+^ responses to axonal stimulation in 3D (Bindocci et al., 2017), the system is a fast raster-scanning two-photon microscope, Bruker Ultima IV *in vivo* system, equipped with an 8 kHz resonant galvanometer (for fast x-axis scan) and piezoelectric objective actuator (for fast z-axis positioning). As is typical for most two-photon setups, volume imaging is accomplished by a consecutive raster scanning of every voxel (at a corresponding position x, y, and z) through time (t), while the speed improvement is traded off for a greatly decreased voxel dwell time. The resulting signal loss is partially compensated with the use of sensitive GaAsP detectors and space-time filters.

**Figure 2:**
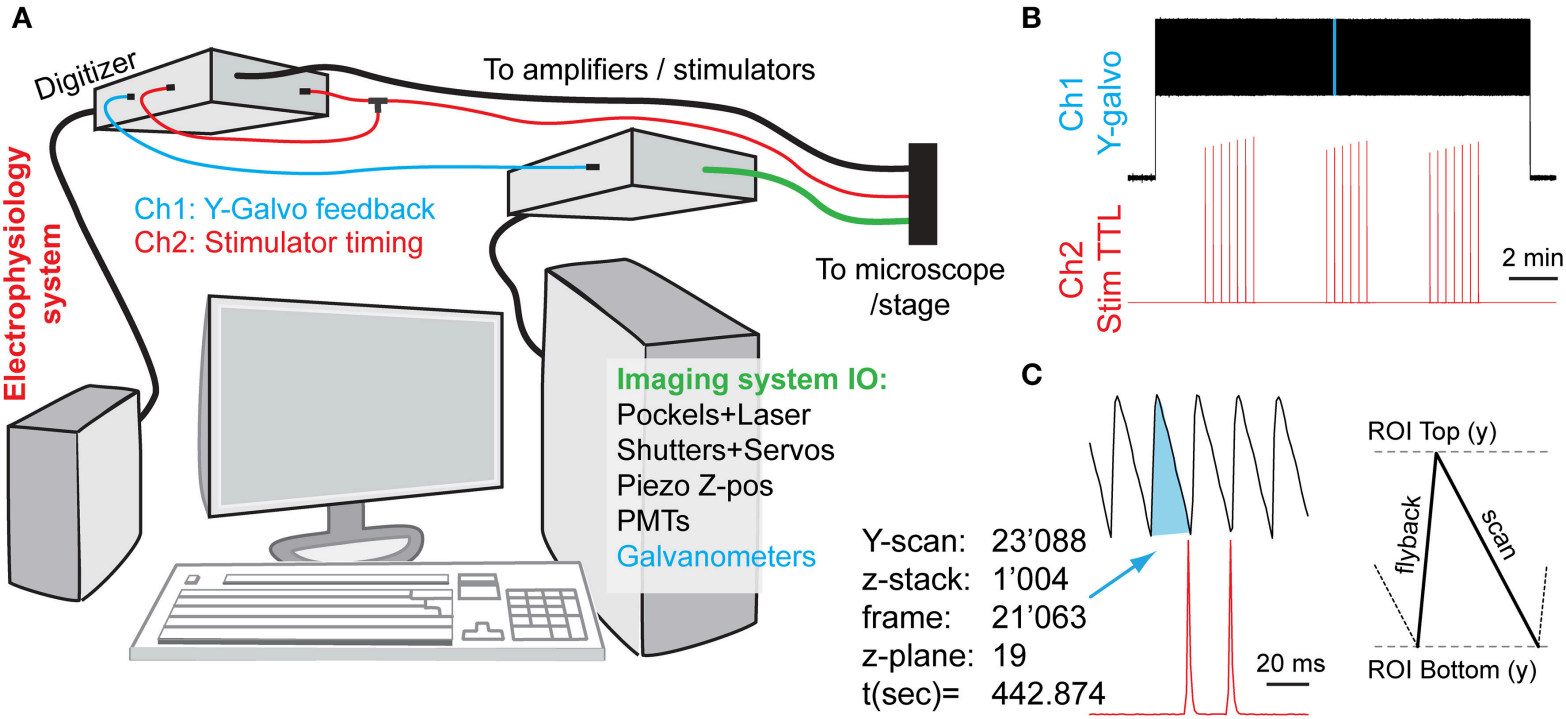
Synchronization of imaging and electrical stimulation via simultaneous capture of timing information from the two systems. By concurrently recording the image frame counts and the electrical inputs, one can later link analytically the imaging and electrophysiology data. **(A)** Schematic diagram of connections. An electrophysiology computer is also simultaneously recording two signals for synchronization: a Y-galvanometer position feedback pin, and a split-off of a stimulator TTL trigger. **(B)** Low-zoom overview of the captured synchronization signals (light-blue highlight is magnified in next panel, not to scale). Vertical deflections in the Y-galvo trace correspond to individual Y-frame scans, whereas spikes on the *Stim TTL* trace indicate the relative timings of axonal stimulation **(C)**. A high-zoom version of the highlighted stretch in **(B)**, showing the relationship between the imaging frame position and the stimulation signal timing. Each Z-stack consumes an entire Y-scan *frame* per focal plane (here 21) plus any additional overhead, depending on whether bi-directional z-scanning is implemented, etc.

*Electrophysiology system* or module: in general, this can range from a simple solution (stimulator TTL pulses from a serial port of a computer, or an embedded platform such as Pic32, Arduino, or Raspberri Pi) to a full-featured electrophysiology suite. In our case, we used an Axon Instruments setup (Molecular Devices): DigiData 1440 digitizer, MiniDigi Miniscope datalogger, MultiClamp 700A amplifier, and a WPI constant-current stimulator. Frame positions and stimulator commands were continuously recorded by MiniDigi (Figure 2A).

*Microelectrode positioning system*/micromanipulators: in our case we used a Luigs & Neumann SM-5 system equipped with an appropriate electrode holder rod, wires, etc.

The procedure for recording correlated electrical and visual data is described below.

#### Axonal electrical stimulation protocols

Experiments involving axonal electrical stimulation are generally performed in brain slices. A slice is placed in the microscope slice chamber equipped with an appropriate aCSF perfusion system and environmental controls. The cells of interest are visually located, the stimulation electrodes placed, and the stimulation/acquisition performed according to the protocol. In our case, we chose to stimulate the axons over an interval of 15 min (see Figure 2B). The protocol consisted in three periods of sparse stimulation, each one lasting 2–3 min (0.05 Hz, or a total of 15 stimulation episodes), separated by intervals without any stimulation of similar duration (2–3 min). This protocol was chosen to reliably separate high background spontaneous Ca^2+^ activity from true evoked activity. Importantly, the electrophysiology and imaging systems used in these experiments need to be accurately synchronized for future data analysis offline, notably for establishing the correct temporal sequence of neuronal activity and astrocytic responses. Alternatively, more targeted stimulation approaches, such as paired whole-cell patch clamp experiments or optogenetic (ChR2, etc.) stimulation could be used to activate the axons in a more spatially restricted manner.

### Synchronization of imaging and electrophysiological recording systems

For analyzing the temporal relationship between electrical and Ca^2+^ activity recorded in the brain preparation, the electrophysiological and imaging data streams need to be precisely synchronized. The exact method of accomplishing the synchronization is highly hardware- and configuration-dependent, and may even be provided out-of-box by the modern imaging systems (albeit generally not designed with electrophysiologists in mind, and thus missing important features).

In our case, we used two different computer systems to perform electrophysiological and imaging recordings, which were synchronized by the electrophysiology computer that simultaneously recorded the timetags from both datastreams. We continuously recorded both systems' state signals on two different channels: (a) the electrical stimulator control voltage (TTL) trace produced by the electrophysiology setup, going to +5 V during the stimulation activation, and (b) the Y-galvanometer feedback position trace output from the imaging setup. In practice, this involved the continuous capture of both channels at 1 kHz sampling rate using MiniDigi 1A digitizer (Molecular Devices) and the consequent comparison of the two resulting signals (see Figure 2). Slow MiniDigi was used in order to free up the much more powerful and faster (up to 250 kHz) Digidata1440 to orchestrate the stimulation and recording experiments. Importantly, even using the MiniDigi with such low sampling rate, the exact stack location (t, z) of each imaging frame, and to some extent even the line position within a frame (y) that corresponded to the electrical stimulation of the axons, could be unambiguously identified (see Figure 2C). More precise x-y coordinates of each voxel, if needed, could be estimated within each frame by sampling the galvanometer position signals at a faster rate that corresponds to the pixel dwell time (variable, on the order of 0.1 μs in our setup), or, ideally, by acquiring a pixel clock signal. More generally, *Frame out, Pixel clock*, or other related imaging system outputs could be captured for similar purposes.

Importantly, care must be taken to ensure that sub-millisecond stimulus events (typically lasting 50–100 μs) are adequately captured by the digitizer that may operate at a much slower sampling rate. In our testing, MiniDigi 1A, while limited to a maximum 1 kHz “*gap-free*” continuous sampling rate (1,000 μs), also provided two software-configurable filter options: analog or min/max filtering. Notably, either one of these filters permits reliable detection of the pulses originally delivered at 5 V and lasting tens of microseconds. The resultant stimulation events appear to last 2–3 sampling points (milliseconds), with a maximum peak amplitude of only 0.1–2 V, yet always greatly above the noise level of the baseline (see Figure 2, but note the sub-sampling artifacts in ramping amplitudes in B). The min/max filter option provided the highest amplitude of peaks, but also occasionally introduced some artifacts, and therefore the analog filter option was chosen.

Where available, it would also be prudent to store a time-stamped copy of the electrophysiology signal on the imaging system. As an alternative, the amplified traces can also be saved as a new image channel, albeit with some data loss between frames and during the galvanometer/piezo fly-back intervals, and with decreased fidelity or range (imaging cards typically have a lower ADC resolution of 8–12 bits vs. 16+ bits typical for the standard electrophysiology equipment).

### Ca^2+^ recording procedure: surgery, slice preparation, fiber targeting, and recording of Ca^2+^ transients

#### Craniotomy and viral labeling of axons (optional)

Tracing axonal projections requires reliable but sparse labeling of neurons, which can be accomplished by using, e.g., Thy1-driven mouse lines with fluorescent neurons, or by the stereotaxic injection of tracer dyes or viral constructs in other lines not expressing fluorescent neurons. In our case, for labeling medial perforant path projections we have used the second strategy, i.e., stereotaxic injection of adeno-associated viruses into the entorhinal cortex to express fluorophores (either membrane tdTomato IX or the red GECI, jRCaMP1a/b; Bindocci et al., 2017) in neurons. A detailed protocol for performing viral injections can be found elsewhere (see e.g., Jiang et al., 2014). Briefly, we anesthetized the mice with isoflurane, drilled a small opening in the scull and used a pulled glass capillary to inject a small volume of the virus (0.5–1.0 μl at viral titer of ~1 × 10^12^ VG/ml) stereotaxically at the following coordinates (bregma: −4.5 mm; lateral: 2.85 mm; ventral: −3.30 mm, adjusted slightly for different mouse strains and ages) over a period of 5–10 min. After an additional 10 min in which the capillary was left in place to permit viral diffusion, the injector was withdrawn, the craniotomy was closed up, and the mice allowed to recover. Analgesia was provided by paracetamol (500 mg/250 ml) in the drinking water starting 1 day prior to the surgery and for several days afterwards. Good viral expression was apparent 4–5 weeks post-infection. All procedures have received ethics approval and were conducted under license and according to the regulations of the cantonal Veterinary Offices of Vaud, Switzerland.

#### GECI expression in astrocytes

Expression of the GECI, GCaMP6f (cytosolic or membrane-bound) in astrocytes can be obtained by using transgenic mice carrying the floxed GCaMP6f gene (Jackson 024105) crossed with mice expressing inducible Cre under an astrocyte promoter, such as human GFAP (GFAP-CreERT2, Hirrlinger et al., 2006). Expression of GCaMP6f in astrocytes is triggered by tamoxifen intraperitoneal injections in the mice (Tamoxifen in corn oil, 10 mg/ml, injected i.p. at 0.1 ml/10 g body weight) and normally requires about 2 weeks from the start of the injections. A large number of injections (8 for 8 days) leads to GCaMP6f expression in a large fraction of the astrocytes, whereas a more limited number of injections, typically 1–3 over 5 days, allows for expression of the Ca^2+^ indicator in a restricted number of astrocytes and is more appropriate when detailed single-cell studies are performed. Thus, selection of a GCaMP6f-positive astrocyte without any other GCaMP6f-positive astrocyte in the surroundings, allows for unambiguous identification of the Ca^2+^ activity as belonging to that specific astrocyte, particularly when recorded from sub-resolved regions like the gliapil. Alternatively, viral constructs of GCaMP6f and other GECI under specific astrocyte promoter (Shigetomi et al., 2013) are available and lead to expression of the indicator in astrocytes upon intracranial (stereotaxically guided) virus injection in the brain region of interest. In our experience, this procedure may require at least 6–8 weeks to produce a physiological Ca^2+^ activity in the astrocytes, whereas at shorter timings the transients have distorted kinetics, possibly due to a still reactive state of the astrocytes upon the injection.

#### Slice preparation (optional)

Acute brain slices allow for superior microscopic observation and manipulative access to the tissue, but *in vivo* imaging (with additional steps required to ensure optical access, such as cranial window preparation) can offer a large number of advantages, including a potentially much longer window of sample viability. In our studies of the hippocampal dentate gyrus, we used acute horizontal brain slices prepared from 2 to 4 month old mice expressing fluorescent tracers in their axons [see section Craniotomy and Viral Labeling of Axons (optional)] and GECI in astrocytes (see section GECI Expression in Astrocytes; Bindocci et al., 2017). Briefly, the animals were anesthetized with isoflurane and decapitated. The brain was rapidly extracted, placed into an ice slurry solution (regular aCSF with half of NaCl substituted by sucrose, carboxigenated with 95% O_2_/ 5% CO_2_), dissected, and glued to the stage of the microvibratome (Leica VT1000 or Microm 650). The slices were incubated in aCSF at 34°C for at least half an hour. To label the astrocytic core structure, we added a brief incubation in low-concentration sulforhodamine 101 (5 min in 0.05 μM SR101) followed by a washout step of at least 15 min.

#### Axonal fiber targeting and stimulation

For electrical stimulation, the labeled axons should be approached with a patch pipette. Because the individual fibers are generally thin and have weak fluorescence, it is advantageous to use micromanipulators equipped with a numerical readout of the electrode position. Exact location of the fiber can be noted down, and the electrode mostly pre-positioned by dead reckoning. Because there is some mismatch between the Dodt and two-photon fluorescence planes, it may also be advantageous to label the outside of the glass electrode with a fluorescent reporter such as quantum dots (Andrasfalvy et al., 2014). In our studies, we always stimulated at a minimal distance of 40 μm from the astrocyte crossed by the labeled axons (see discussion in Introduction). Stimulation protocols were as detailed in section Axonal Electrical Stimulation Protocols and 3D Ca^2+^ imaging of astrocytes was performed by recording at ≥2 Hz stack rate in cuboids of about 20–30 xyz focal planes (Bindocci et al., 2017).

### Data handling, exploration, and analysis

In this section, we describe the main steps of treatment of the data acquired during the experiments. At first, data handling involves conversion and storage of the data files (image series and electrophysiological recordings) for later, off-line analysis as needed. Data can be safeguarded on external disks or network attached storage (NAS) computer systems. Next, data exploration typically involves loading and filtering the data, and displaying them for the user in an on-line interactive manner. Two of our plugins are designed to allow 5D filtering and interactive multi-ROI placement and associated signal display. Finally, data analysis involves extracting signals from defined populations of cells, regions of a cell, or voxels. One of our plugins is designed to automatically identify the size of reliably-responding regions that exhibit consistent increases in Ca^2+^ transients in response to axonal stimulations.

#### Volumetric data storage and access within ImageJ framework

ImageJ software (NIH) (Schneider et al., 2012) provides a free and open framework for image data storage, retrieval, processing, and display that over decades has become one of *de facto* standards for scientific image analysis. Modern versions of ImageJ natively allow storage of multi-dimensional imaging data via the Hyperstack functions. Users can define up to five dimensions of the hyperstack as follows: width (X), height (Y), number of channels (C), number of slices (Z), and number of time-frames (T). In addition, they can control a number of associated properties, such as display mode, underlying voxel data type (8, 16, or 32 bits, etc.), and a number of metadata properties such as slice labels and scales. This is fully sufficient for a user-friendly handling of the storage, retrieval, and programmatic analysis of the multi-color volumetric data acquired through time such as the multi-color 3D movies of spontaneous and evoked astrocytic Ca^2+^ activity (see e.g., Bindocci et al., 2017).

Programmatically, the 5D voxel data can be accessed via either a *Macro* script (user-friendly, and very handy for chaining built-in commands and plugins, but generally too slow for extensive voxel-wise analysis), or natively through Java plugins (requiring larger programmer participation, but resulting in several orders of magnitude speed improvements during computations). Using either method, extraction of the data for a voxel at coordinates (x, y, z, t, c) is accomplished by locating and extracting a single 2d (X-Y) frame in the stack that corresponds to the desired z, t, and c coordinates. For ease of access (but at a cost of doubling memory requirements), two of our three plugins first transfer these data into a structured 5D memory array (e.g., buff CTZXY [c][t][z][x][y]). The only practical limitation of such approach is when the data size exceeds the amount of RAM available to Java VM, or when any of the five array dimensions exceeds the maximum value for the Java integer type (obtained via Integer.MAX_VALUE parameter), currently equal to 2^31^-1, or about 2 billion.

#### Filtering (Gaussian_Filter5D plugin)

After the data stacks are converted and loaded, the data can be processed with filtering as usual. Because the built-in tools are unable to perform filtering over 2 dimensions on stacks containing both T and Z series, we have generated a 5D filter plugin able to perform the filtering on 5D data in up to 4 dimensions (x, y, z, and t) simultaneously. The ability to pre-subtract a noise floor from the data (thresholding, or *DC offset*) is also provided. This is useful to remove low-level electrical noise pickup (e.g., 50/60 Hz) from the recordings.

Here below is shown the sample user interface for the *Gaussian Filter 5D* plugin:

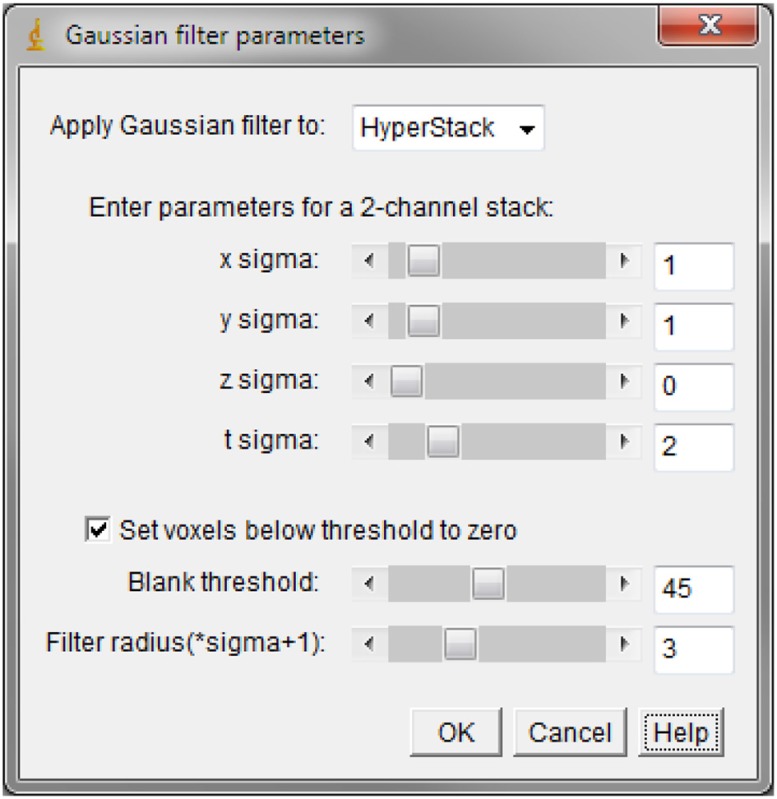



#### Data exploration (MultiROI TZ profiler plugin)

Once processed, data can be manually explored by placing a user-generated ROI and displaying the corresponding Ca^2+^ trace. Recent versions of ImageJ have added the ability to move interactively and resize the ROI while getting an instantaneous update of the trace graph. We have further expanded this ability by adding the following capabilities: (1) simultaneous placement of multiple ROIs and display of the corresponding Ca^2+^ traces; (2) normalization of each trace to the min/max values; (3) overlay of the original trace with a filtered trace; and (4) simultaneous display of the externally loaded stimulus file on top of the Ca^2+^ traces (see section Expected Results below).

Here below is shown the main interface for the *MultiROI TZ profiler* plugin:

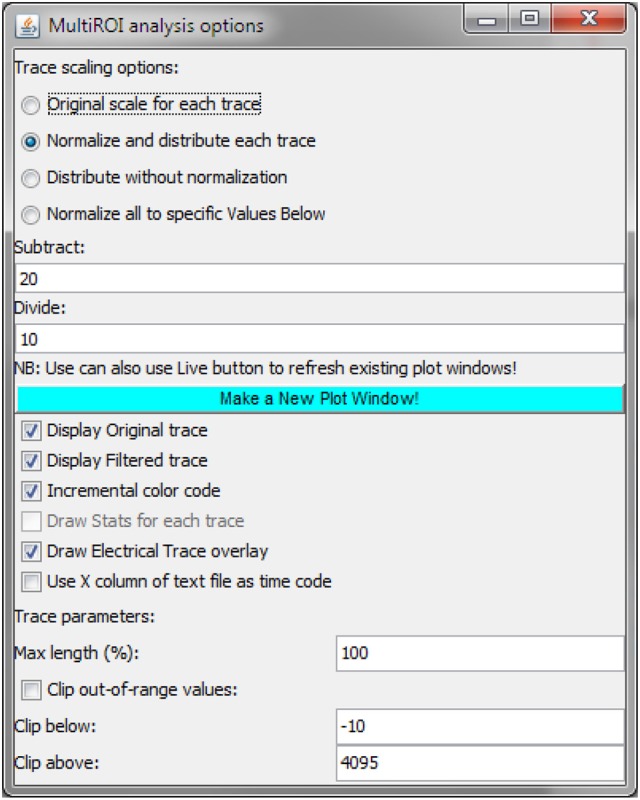



#### Data analysis using correlation (correlation calculator plugin)

Correlation-based analysis approaches provide a useful means of detecting broadly co-incident patterns in the data. The correlation method has been successfully used in automatically building *unitary* ROIs (e.g., corresponding to neuronal somas) based on the expectation that neighboring pixels belonging to the same underlying structure exhibit Ca^2+^ transients with fundamentally similar kinetics. Detecting and grouping closely correlated pixels allows for the build-up of a larger ROI with vastly improved SNR. The correlation-based techniques can also be used to rapidly screen for time-locked responses in the data. This is the approach that we recently used to identify the regions of an astrocyte that respond to minimal axonal stimulations: the task was severely complicated by the fact that astrocytes also display endogenous Ca^2+^ activity, that the reliably responding regions are small, that the response properties are variable, with a large jitter in response delay, on-kinetics and magnitude. The work-around for these difficulties was to perform correlation analysis on the Ca^2+^ signal from each individual voxel using repeated trials of stimulation, looking for signals that occurred consistently in each trial within a given time window after each stimulation. With this approach, the endogenous or low-probability responses that occurred by chance within the imposed time window in some of the trials were canceled out, while the repeated Ca^2+^ increases (even with variable delay/waveform) were kept. We called this approach “*normalized cumulative cross-correlation*” because, mathematically, the computation was performed by calculating a cross-correlation of the normalized fluorescence Ca^2+^ trace with an externally loaded (optionally *binarized*) stimulus file (Bindocci et al., 2017). The resulting correlation coefficients were added up for all values of time lag between 0 and a pre-defined time window (corresponding to 2.5 or 5 s in our case). In practice, this allowed for a direct, user-independent quantification of the estimated active volumes or responding regions. During the analysis using the plugin, the user can specify the time size of the correlation window used to detect the response (in t frames) and the size of an X-Y filter (averaging neighboring pixels) that can be applied to the data. The time size of the correlation window will vary depending on the sampling rate, the expected time delay between the stimulation and the response, and, to some extent, on the indicator used. This window size also acts as a normalization window for the fluorescence trace.

Here below is shown the user interface for the *Correlation Calculator* plugin:

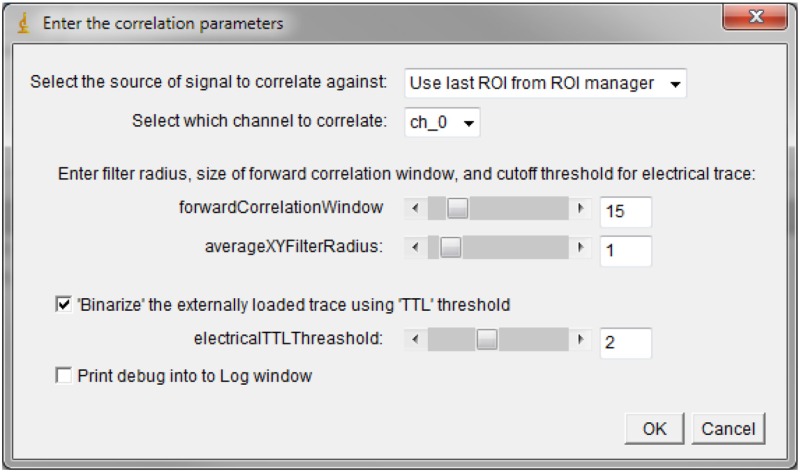



## Expected results

About 5 weeks after viral injection, neurons are expected to start expressing the selected fluorophores at quantities sufficient to visualize individual axons. The exact mode of visualization will depend on the fluorophore and the imaging technique used, but in general, it may be better to choose red-spectrum fluorophores (such as tdTomato as a morphological dye, or jRCaMP as a Ca^2+^ indicator) due to a somewhat lower tissue autofluorescence in the red spectrum. The figures below show the typical appearance of labeled axonal fibers in slices, and a typical example of electrically-evoked Ca^2+^ transients recorded from the *gliapil* of GCaMP6f-expressing astrocytes.

## Discussion

In this manuscript, we provided a practical guide to our recently developed approach to study astrocytic responses to neuronal activity by means of 3D two-photon Ca^2+^ imaging, astrocyte-specific GECI expression and axonal fluorescent labeling. We also presented the software tools *ad hoc* developed for this study. We release these tools for the community in the hope that they prove useful also to others. In this sense, the current article is intended to act as a paper of record (citation) for these software programs. Their most recent versions including bug fixes can be obtained from the Volterra Lab webpage at https://wwwfbm.unil.ch/dnf/group/glia-an-active-synaptic-partner/member/volterra-andrea-volterra.

### Conceptual advances of 3D Ca^2+^ imaging over 2D imaging

Astrocytes are highly three-dimensional cells that show a wide spectrum of Ca^2+^ activity consisting of transients of varied features mostly occurring at unpredictable times and spatial locations in sub-portions of the total 3D astrocytic structure. Consequently, studies of this activity done with conventional 2D two-photon imaging turn out to have several relevant problems. Thus, we estimated that even an optimally placed two-photon focal plane will capture only about 5% of the total astrocyte volume, and about 10% of its total activity, and even that incompletely (Bindocci et al., 2017). Moreover, unlike in neurons, the activity easiest to capture, i.e., that seen in the somatic region of the astrocyte, is very infrequent compared to the activity observed in processes and peripheral regions of the cell, and cannot be taken as representative of the overall cell activity or output. Figure 1 illustrates the problems of 2D imaging, overcome by 3D. Firstly, with 3D, much more of the cell is captured and analyzed (Figures 1A,B). Secondly, individual planes no longer split a single contiguous structure into multiple fragments, therefore allowing unambigious distinction between *actual* functional microdomains (small regions of the astrocytes that act biologically as a unit), and *false* microdomains, small active regions artificially generated by the experimenter by focusing on an individual focal plane, cutting *de facto* structures that extend three-dimensionally above and below the selected plane (Figure 1B, red mask). Thirdly, events lying totally or partially outside of the selected focal plane are not missed as instead occurs with 2D imaging (Figure 1B), allowing correct calculations of the events frequency and spatial-temporal properties. The software provided here allows to optimize the analysis workflow on the multi-dimensional datasets.

### 3D imaging and time-correlation analysis capture the local astrocytic responses to minimal axonal stimulation

**Figure 3:**
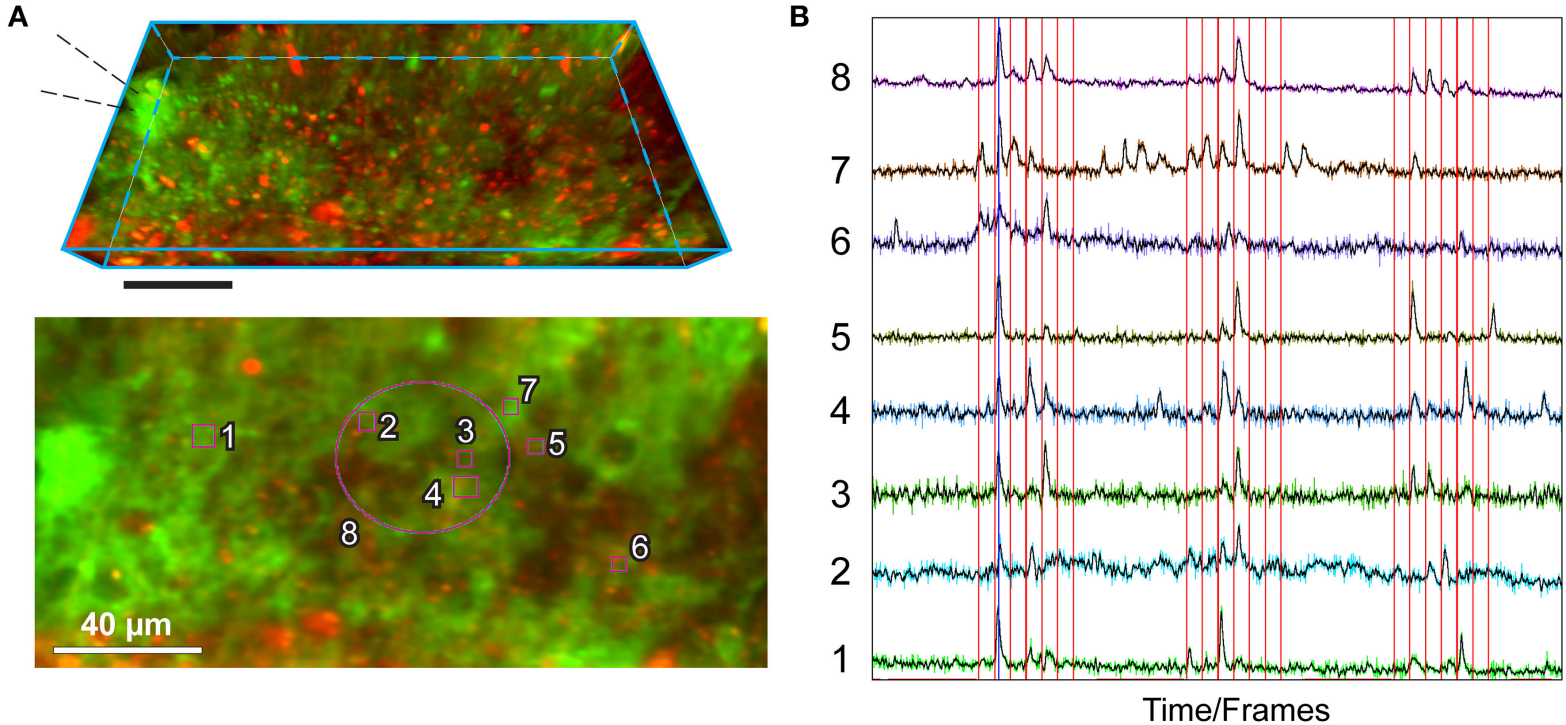
Example of utilization of the *MultiROI TZ Profiler* plugin: production of normalized Ca^2+^ traces extracted from multiple interactively placed ROIs. The figure shows representative results obtained by performing sparse axonal stimulations in the cerebellar molecular layer, visualized with the *MultiROI TZ Profiler* plugin. **(A)** *Top*: Side view of a TZ stack (21 focal planes spaced at 1 μm each); astrocytes are transgenically expressing GCaMP6f (green) and are also co-stained by a brief application of SR101 (red) for 5 min. Scale: 40 μm. The stimulation pipette is placed to the left as indicated. *Bottom*: Relative placement of eight ROIs used to extract the signals in **(B)**, overlaid on top of the average TZ projection of the above stack. **(B)** Real-time readout of the fluorescence obtained from each of the ROIs, as labeled. The electrical stimulation train (interactively loaded from an ASCII-based columnar text file) is overlaid in red. A running average of each colored trace is displayed in black. Traces interval corresponds to 1,200 time-frames (complete Z-stacks) acquired in 440 s. Stimulation protocol as in (Bindocci et al., 2017) but sped up twice (Savtchouk et al., 2016): single pulse or paired/train stimulations (100 Hz) are delivered once every 10 s in the following sequence: 1, 2, 3, 4, 5, 1, 2 pulses. The three stimulation epochs are separated by quiescent intervals.

Compared to prior research methods employed in the field, our current region-agnostic approach requires no assumption regarding spatial size and location of the astrocytic responses (i.e., the size and placement of the ROI/VOI for signal extraction). Therefore, it provides a good option for unbiased detection of the smallest noise-limited responses and their analysis. For analysis, we presented our software tools allowing for both automated detection of responding regions and, in an interactive version, for real-time extraction/visualization of the signals with a manually placed ROI (Figure 3). The use of these tools, together with voxel-level analysis of a whole astrocyte imaged volume, allowed us to identify small compartments, generally located in the fine gliapil meshwork, that reliably respond to repeated sets of minimal neuronal inputs, and are suppressed when the stimulations are performed in TTX (Bindocci et al., 2017). Such compartments likely represent local units of astrocytic excitation in response to sparse firing of one or few axons. Their volume corresponds to a very tiny fraction of the astrocytic volume, 1% at most, and their location cannot be easily predicted *a priori* in view of the tortuous axonal paths, at least in the hippocampal dentate gyrus (see below). These experimental issues make such compartments practically impossible to identify with the investigation approaches used until now, notably with conventional single-plane (2D) two-photon imaging. Most likely this is also the reason why several such studies could see an astrocytic response in their randomly selected focal plane only upon stimulation of multiple axons with high current strength, which led them to conclude that astrocytic activation occurs only in response to intense neuronal activity. Additional issues complicate identification of the responding compartments, including the large amount of overlapping endogenous Ca^2+^ activity seen in the astrocytes and the variable delays observed between axonal stimulations and evoked astrocytic responses. All the above issues were overcome by our approach of imaging large volumes of astrocytes and performing multiple rounds of stimulation to successfully separate truly evoked, reliable responses, from spontaneously coincident noise (Bindocci et al., 2017). On balance, high-resolution whole-volume imaging and voxel-by-voxel automated analysis provide a very promising venue for future understanding of astrocytic and neuronal computation during spontaneous conditions, as well as during learning and behavioral tasks.

### Differences in axonal properties and organization influence the study of the astrocytic responses to neuronal inputs

**Figure 4:**
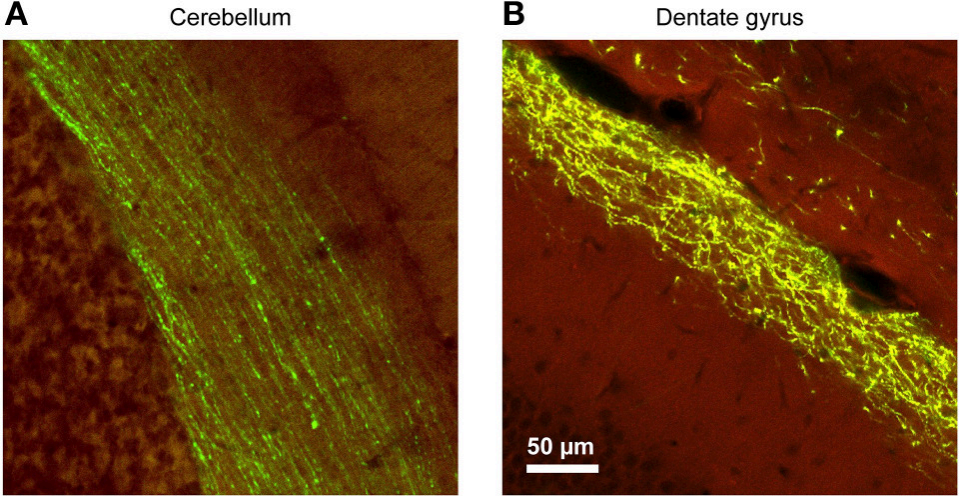
The morphological characteristics of the axons and the trajectories of their paths vary considerably between brain regions, requiring careful adjustments of the stimulation parameters. In this example, YFP-expressing adeno associated virus (AAV) was injected into the brain of mice at various locations, and the resulting axonal labeling was visualized across the different brain regions. **(A)** Parallel fibers of the cerebellar molecular layer show remarkable linearity, maintaining their direction and position for hundreds of micrometers. These properties greatly facilitate axonal stimulation experiments. **(B)** In contrast, Perforant Path projections in the dentate molecular layer of the hippocampal formation show a large degree of tortuosity and deviation. While the general layer identify is maintained, position of individual fibers cannot be predictably extrapolated. Therefore, focal stimulation of several adjacent axons will invariably diverge with increasing distance from the pipette [Virus labeling data taken from Allen Brain Institute dataset (Oh et al., 2014), used with permission].

When looking for the astrocytic responses to axonal stimulation, there are other sources of variability that need to be carefully considered, like the inherent differences in axonal properties and organization in different brain areas and circuits. As discussed above, some axons greatly deviate from the mentally projected paths assumed by the experimenter (thus moving the astrocytic responding regions away from the imaged focal plane), and others not. In Figure 4, we show two extreme situations observed in two different brain regions: on the one hand, the tight beams formed by the extremely thin, straight, unmyelinated axons of the cerebellar granule cells (Figure 4A) and on the other, the loosely arranged, thick, inter-twined, and largely myelinated entorhinal cortex projections to the dentate gyrus (Figure 4B). Blind stimulation experiments performed in these two regions will likely give very different results. Activated cerebellar bundles stay tight for hundreds of microns and are much more likely to encounter their glial partners in the same imaging plane (see e.g., Figure 4, and Sullivan et al., 2005). In contrast, in the hippocampus, the activated perforant path fibers rapidly spread out and diverge, making capture of their interactions with the glial partners by a 2D plane much less likely (see above and Bindocci et al., 2017). Attention should be put also to the fact that some axons are myelinated and others not. This translates into a need for different stimulation parameters (pulse strength and duration) to induce action potentials. Thus, the myelin sheath itself can act as a potent dielectric insulator, reducing the direct effects of the currents and the capacitive coupling of the axon to the voltage transients. For instance, local, targeted activation of the parallel fibers (unmyelinated axons) can be achieved by ejecting minimal currents (1–4 μA typical, Sullivan et al., 2005; Savtchouk et al., 2016), while stimulation current needs to be increased by one order of magnitude in the dentate gyrus (Di Castro et al., 2011; Bindocci et al., 2017). Even moving the stimulation electrode by a few μm sideways has different consequences in the two cases, as it will generally drastically increase the failure rate in the cerebellum, but not in the hippocampus. This characteristic of the cerebellar parallel fibers is well-known and exploited in the field (see e.g., Sullivan et al., 2005; Gao et al., 2006; Soler-Llavina and Sabatini, 2006; Abrahamsson et al., 2012; Savtchouk et al., 2016). Several studies have successfully demonstrated the targeted, spatially restricted nature of such stimulation using either Ca^2+^ imaging in the stellate cell dendrites (see e.g., Figure 8 in Soler-Llavina and Sabatini, 2006) or optical detection of extracellular glutamate released upon parallel fiber stimulation (e.g., Figure 2 of Okubo et al., 2010), even when stimulating with very large current strength.

### Variability of the astrocytic responses

Astrocytes and neurons respond differently to information flow. For instance, evoked Ca^2+^ transients are much less likely to follow a predictable, stereotypical waveform and spatio-temporal distribution in astrocytes than in neurons, making them harder to detect. Fewer *a priori* assumptions can be made for studying astrocytic compared to neuronal Ca^2+^ transients, particularly regarding their size and location. For example, contrary to the situation in neurons, the astrocytic soma appears to be of little use for recapitulating astrocytic excitability, as most of the Ca^2+^ activity in astrocytes is local and occurs, not at the soma, but at the cell peripheries, including astrocytic processes, “gliapil” and end-feet. For this reason, 3D-based volumetric imaging approaches appear to be the most promising way to capture and study the full range of astrocytic Ca^2+^ activity. Even though the resulting data complexity is challenging, the 3D signals can nevertheless be amenable to interactive visualization, exploration, and analysis, including with the early tools released by this lab.

### Multi-dimensional data analysis

The problems of imaging and visualizing multi-dimensional data are not unprecedented, and significant progress has been made in the last few years. Multiple comprehensive frameworks exist for handling such data, such as Image J, Matlab image processing, and Bitplane Imaris to name just three that we utilized. Of these, Image J provides an attractive option because it is free and open-source, possesses good speed and cross-platform compatibility with the full source code available in the public domain, is user-friendly, fully customizable and expandable, and is continuously maintained and improved by a team of highly competent experts (NIH). Importantly for us, Image J provided native out-of-the-box support for multi-dimensional image data storage and handling, and already had a functional toolbox of built-in functions in tools. This allowed us to develop three simple plugins that facilitate exploration and analysis of 5D data (see Bindocci et al., 2017 for practical application). Because our tools are implemented natively in Java and take advantage of the multi-threading capability of modern CPUs, they provide good speed, generally exceeding that of other higher-level scripting approaches.

Overall, in writing this practical guide article on our 3D Ca^2+^ imaging approach and in releasing the related analysis tools we hope to have made a concrete step for convincing the scientific community to apply multidimensional approaches to the functional study of axon-astrocyte interactions in view of their superior quality of investigation.

## Author contributions

IS: Wrote the plugins (software) and made the figures; GC: Established the original stereotaxic AV injection for transgenically labeling EC-DG projections; AV: Guided and supervised the study; IS and AV: Wrote the manuscript.

### Conflict of interest statement

The authors declare that the research was conducted in the absence of any commercial or financial relationships that could be construed as a potential conflict of interest.
